# Cost‐effective process for the production of *Monascus* pigments using potato pomace as carbon source by fed‐batch submerged fermentation

**DOI:** 10.1002/fsn3.2496

**Published:** 2021-08-04

**Authors:** Xiaoju Chen, Minmin Chen, Xuefeng Wu, Xingjiang Li

**Affiliations:** ^1^ College of Chemistry and Material Engineering Chaohu University Chaohu China; ^2^ Key Laboratory for Agricultural Products Processing of Anhui Province School of Food and Biological Engineering Hefei University of Technology Hefei China

**Keywords:** fed‐batch, hydrolysate, *Monascus purpureus*, pigment, potato pomace, submerged fermentation

## Abstract

Potato pomace, generated from starch‐processing industry, was applied as a cost‐effective resource for producing *Monascus* pigments via submerged fermentation. First, the pigment‐production capacity of potato pomace and its hydrolysate was compared. The results indicated that potato pomace was superior to its hydrolysate when used for producing *Monascus* pigments. The red and yellow pigments produced in potato pomace medium reached 27.8 and 19.7 OD units/ml in 7 days, with the yield of total pigments at 1,187.5 OD units/g, respectively, increased by 127.9%, 19.4%, and 46.3% compared with the data obtained from hydrolysate. Meanwhile, the citrinin produced in potato pomace medium decreased by 22.6%. Afterward, potato pomace, without hydrolysis, was used as carbon source to obtain the optimal pigment production conditions. In the batch fermentation process, it was found that high amount of pomace inhibited the growth rate of mycelia and the productivity of pigments, and the fed‐batch fermentation process could enhance the yield and productivity of pigments. With the same final amount of pomace (80 g/L), the maximal levels of total pigments and productivity obtained from fed‐batch process reached 118.8 OD units/ml and 13.2 OD units/(ml·day), which presented an increase of 35.2% and 67.1% compared with the not fed‐batch group, respectively. The results demonstrated that potato pomace was a cost‐effective substrate for producing *Monascus* pigments in terms of pigment production capacity and productivity when fed‐batch submerged fermentation was applied.

## Graphical abstract

Potato pomace was evaluated as economical substrate for pigment production. Potato pomace was superior to its hydrolysate for the pigment production. Fed‐batch was an effective process in terms of production capacity and productivity. Supplying a valuable reference for seeking economical natural pigment process.

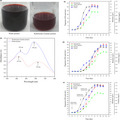

## 1 INTRODUCTION

Pigments can endow products with more attractive characteristics, more appetizing and better sensory experience, and are widely used in beverages, cosmetics, pharmaceuticals, and sweets. At present, the pigments applied in food and other industries are artificial pigments, such as allura red, carmoisine, and tartrazine. However, it was found that long‐time consumption of artificial pigments may be harmful, especially to children (Nawaraj, 2015). By comparison, it was believed that natural pigments generated from plants and microorganisms were environmentally friendly, harmless, and beneficial to human health (Aruldass et al., 2018; Shahid, Salam, & Mohammad, 2013). At present, more people are concerned about their healthy diet, and it is a developing trend that natural pigments replace the artificial pigments. Therefore, the market demand for natural pigments is great in future.

The production of pigments by microorganisms has the advantages of high yield, short productive cycle, nondependent on weather conditions, and good economical benefit. Several microorganisms could synthesize pigments, including *Monascus spp*. (Li et al., 2019; Liu, Guo, et al., 2019), *Penicillium spp*. (Kantifedaki et al., 2018; Wang et al., 2011), yeasts (Alipour et al., 2017), and *Rhodopseudomonas palustris* (Liu et al., 2019). The classic example of pigment‐production microorganisms is *Monascus spp*. (Vendruscolo et al., 2016). It was reported that hundreds of secondary metabolites were produced by *Monascus spp*., among which were mainly red, yellow, and orange pigments, which were added into foods for many years (Chen, Chen, et al., 2017).

Generally, the most known carbon source used for *Monascus* pigment production is rice. Since rice is the staple food of the population in many countries, it is a tendency to reduce the use of rice in *Monascus* pigment production. In order to obtain more economic pigment production processes, several by‐products generated from agroindustry and food industry have been used as substrates, such as apple pomace (Vendruscolo & Ninow, 2014), orange processing waste (Kantifedaki et al., 2018), lactose (Costa & Vendruscolo, 2017), corncob hydrolysate (Zhou et al., 2014), okara (Sun et al., 2020), and sugarcane bagasse hydrolysate (Hilares et al., 2018), and the results demonstrated that by‐products will be a promising carbon sources for *Monascus* pigment production.

As the main food crop, potato is widely planted in the world. In China, the annual production of potatoes is about 5.5 million tons, most of which are used for starch production, and a large amount of potato pomace is generated at the same time. The pomace is usually processed as animal feed or discarded directly. The wet potato pomace will cause serious pollution if discarded directly because of the easy to corruption. Actually, potato pomace is rich in fermentable substances, for instance carbon (starch) and nitrogen (protein) resources. In theory, potato pomace can be used as cost‐effective resource for producing higher value‐added metabolites by microorganisms. However, reports on the production of secondary metabolites from potato pomace by submerged fermentation are few, especially on the production of pigments.

In order to avoid the waste of potato pomace, the present study was performed to evaluate the practicability of using potato pomace and its hydrolysate as cost‐effective resources to produce *Monascus* pigments by submerged fermentation. The favorable conditions for improving pigment production were searched by flasks, batch, and fed‐batch experiments. The results demonstrated that potato pomace, without hydrolysis process, was an effective substrate for producing *Monascus* pigments, and fed‐batch submerged fermentation was a feasible process for improving the *Monascus* pigment production capacity and productivity.

## 2 MATERIALS AND METHODS

### 2.1 Microorganism and materials

*Monascus purpureus* CH01 originated from *Monascus purpureus* CICC 5,016 was used (Chen et al., 2021). *Monascus purpureus* CH01 was cultured at 30℃ for 7 days using the PDA slant medium, afterward was maintained at 4℃ in the laboratory of Chaohu University. Potato pomace was obtained from a potato starch‐processing manufacturer located in Hefei city of China. The wet potato pomace was first dried naturally. Afterward, the pomace was grinded and sieved with 40‐mesh sieve.

### 2.2 Preparation of inoculums

Before a new fermentation process, the microorganism was first cultured on a fresh PDA medium as stated above. Afterward, the spore's suspension was prepared through adding optimal volume of sterile water to make the concentration of spore as 3.5 × 10^6^ spores/ml.

### 2.3 Preparation of hydrolyzed sugar

The mixture contains 100 g potato pomace and 2 g ɑ‐amylase (50,000 u/g), and 500 ml water was adjusted to pH 6.0 and reacted at 70℃ for 60 min. Afterward, 0.2 g saccharifying enzyme (100,000 u/g) was added and reacted at 60℃, pH 4.5 for 60 min. Enzymatic hydrolysate solution and residual cellulose were separated by filtration after boiled for 5 min to extinguish enzymes. Afterward, the residual cellulose was hydrolyzed according to the method of Li et al. (Li et al., 2017). The main hydrolysis process is as follows: The residual cellulose was soaked into the 1.8% (w/v) H_2_SO_4_ solution with the mass/volume ratio as 1:10 and hydrolyzed at 220℃ for 5 min. Afterward, excess calcium hydroxide was added to neutralize the hydrolysate. The hydrolysate solution and residue were separated by filtration, and activated carbon was added into the separated hydrolysate for detoxification. At last, the above‐mentioned hydrolysates were combined to obtain the hydrolysate sugar solution of potato pomace.

### 2.4 Fermentation medium and conditions

Unless otherwise stated, the basic submerged fermentation medium and conditions were consistent as follows: per liter fermentation medium contained substrate 40 g, peptone 5 g, KH_2_PO_4_ 0.4 g, MgSO_4_ 0.5 g, FeSO_4_ 2 mg, and ZnSO_4_ 2 mg. The initial pH was controlled at 6.0 by adding acetic acid solution (2 mol/L). The flask (250 ml) experiments were carried out to seek the optimal process parameters. The volume of medium in flask was 100 ml, after inoculated with 10 ml of spore's suspension, and the flask was incubated in the shaker at 28℃, 200 rpm for 7 days. Experiments were first designed to compare the pigment production capacity between potato pomace and its hydrolysate sugar. After the more effective substrate was confirmed, the effects of nitrogen sources (peptone, beef extract, and so on, 5 g/L), C/N ratio, incubation temperature (25–35℃), inoculum size (1%–15% (v/v)), and initial pH (3.0‐8.0) on pigment production were investigated.

The batch or fed‐batch experiment was performed in a 5‐L tank (SY‐3000, Shiyuan, Shanghai, China). The initial volume of medium was 3 L, the inoculum size of spore's suspension was 10% (v/v), and the initial pH was 6.0. The fermentor was operated at 28℃, 200 rpm, and aeration of 300 L/h. For fed‐batch process, the 300 g/L of potato pomace was used as the feeding medium. The sterilization conditions for the flasks and fermentor were at 118℃ for 20 min.

### 2.5 Pigment estimation

The fermented broth was first centrifuged at 7,155 *g* for 12 min. The supernatant was used for estimating the extracellular pigments, and the centrifugated deposit was collected for determining the biomass. The method for the determination of pigments was according to reference (Lv et al., 2017). 1 ml of centrifugated supernatant was mixed with 9 ml of 70% (v/v) ethanol. After rotated at 50℃ and 150 rpm for 30 min, the sample was detected to obtain the maximum absorption wavelength and absorbance by a spectrophotometer (TU‐1901; PERSEE, Beijing, China). The production of pigments (OD units/ml) was expressed as the absorbance units multiplied by a dilution factor. Production of total pigments was the sum of individual pigments. The productivity (OD units/(ml·day)) was defined as the total pigments produced every day in per unit volume. The yield (OD units/g) was defined as the total pigments produced by every gram of substrate consumed. When potato pomace was used as substrate, the yields were calculated based on the hypothesis that all potato pomace was consumed completely, because it was difficult to determine the contents of pomace in the residual solids.

### 2.6 Biomass estimation

The centrifugated deposit obtained above was used to estimate the biomass. When the hydrolysate was used as the substrate, the dry mycelia weight was weighed according to previous study (Tseng et al., 2010). When the potato pomace was used as substrate directly, the dry mycelia weight cannot be obtained since the deposit was a mixture of mycelia and unutilized pomace. Therefore, nucleic acid in the deposit was extracted using 5% trichloroacetic acid (v/v) solution to estimate the biomass, and the process was performed according to the method described by Liu (Liu et al., 2000).

### 2.7 Sugar and citrinin estimation

The ultra‐high‐performance liquid chromatography (ACQUTIY UPLC H‐Class plus, Waters, USA) equipped with a scattering detector was used to analyze the sugar concentrations, and the specific detection conditions were as described previously (Chen et al., 2017). The citrinin concentration was determined using a dual diode array detector, and the method was referred to the published report by Sun et al (Sun et al., 2020). The ACQUTIY UPLC BEH Amide 1.7 μm Carbohydrate Analysis C18 column (100 × 2.1, Waters, Ireland) was used for the analysis of sugar and citrinin.

### 2.8 Statistical analysis

Each measurement was carried out in triplicate. The results were analyzed statistically using the Origin 7.5 software and presented as the mean ± standard deviation.

## 3 RESULTS AND DISCUSSION

### 3.1 The pigment production capacity of potato pomace and hydrolysate sugar

**FIGURE 1:**
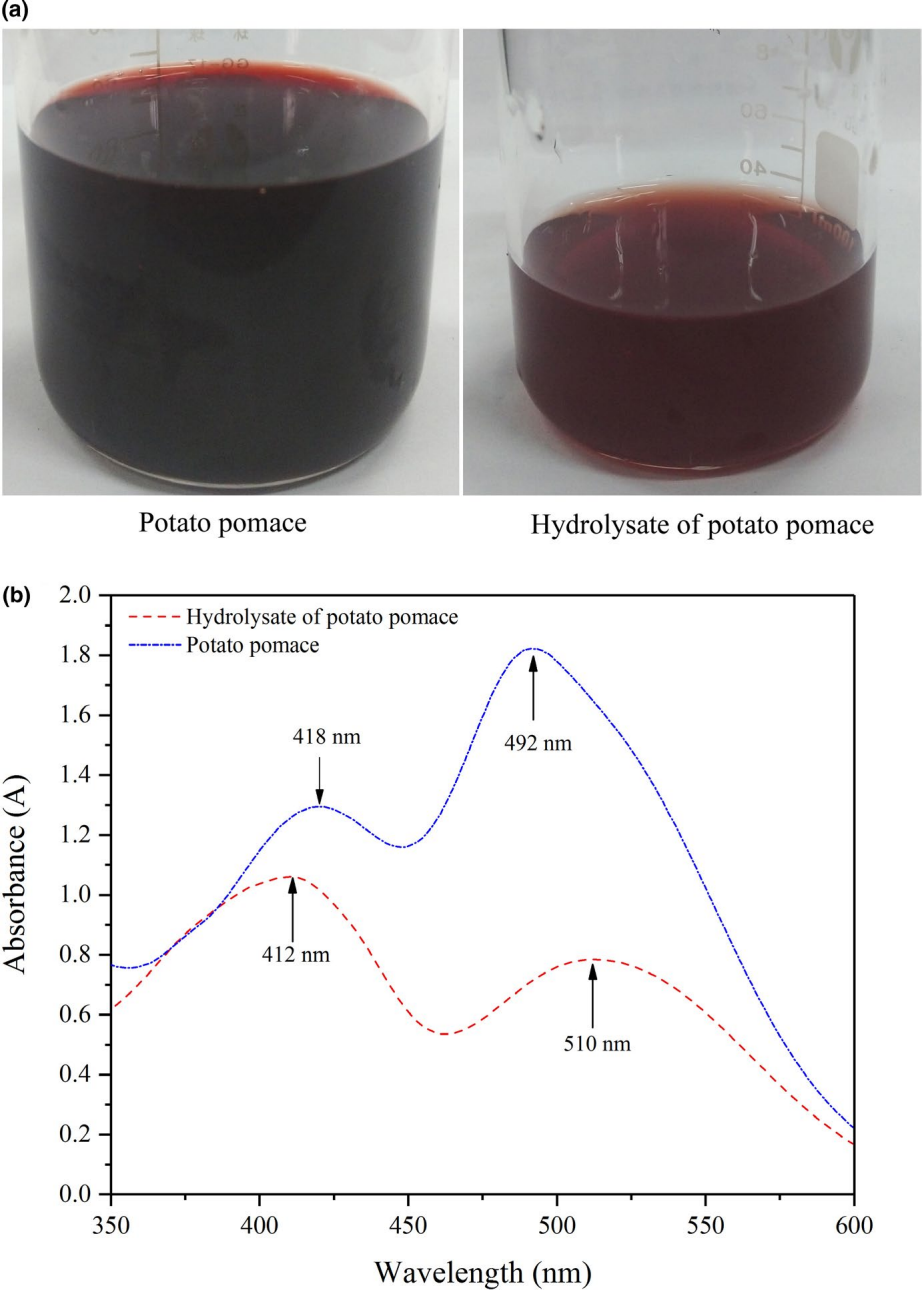
Comparison of the broth color (a) and the scanning spectrum (b) of potato pomace and its hydrolysate

*Monascus* pigments are natural safety colorants and have a bright future for the application in food manufacturing industry. The *Monascus* pigment production process using rice as carbon source has a long history in East Asia. At present, rice is still prevailed in industrial preparation of *Monascus* pigments. In order to save food resources, it is necessary to seek a cost‐effective carbon source for producing pigments, for instance the wastes generated during food manufacturing. In this part, potato pomace and its hydrolysate were used as the substrate to evaluate their pigment production capacity by submerged fermentation, respectively. The results in Figure 1 indicated that both potato pomace and hydrolysate presented the capacity for producing pigments by *Monascus purpureus*, but the phenomena had obvious distinctions. The color in the fermented broth of potato pomace was darker than that of hydrolysate (Figure 1a). In Figure 1b, the UV scanning spectrum analysis results demonstrated that there were principally two characteristic absorption peaks in the wavelength ranges of 495–500 nm and 412–418 nm, respectively, which represented red and yellow pigments (Yang, Liu, et al., 2015). For the broth of potato pomace, the maximum absorption wavelengths for red and yellow pigments were 492 and 418 nm, respectively. While for hydrolysate of potato pomace, the maximum wavelengths for red and yellow pigments were 510 and 412 nm. The peak at 492 nm was steeper than the peak at 418 nm when using potato pomace as carbon source, but the phenomenon of hydrolysate was on the contrary, which suggested that the yield and ratio of pigments were different. In following experiments, the production of red and yellow pigments was estimated at the wavelengths of 510 and 412 nm for hydrolysate, while the red and yellow pigments produced from potato pomace medium were measured at wavelengths of 492 and 418 nm.

Generally, glucose was supposed as the preferable substrate for producing *Monascus* pigments (Lee et al., 2001), but this phenomenon was not observed in this study. Although most of sugar in hydrolysate was glucose, the pigment production capacity was inferior to potato pomace with no hydrolysis. As shown in table 1, the production of red and yellow pigments obtained from potato pomace reached 27.8 and 19.7 OD units/ml in 7 days, with yield of total pigments as 1,187.5 OD units/g, respectively, increased by 127.9%, 19.4%, and 46.3% compared with the data obtained from hydrolysate. It was found that the ethanol concentration in hydrolysate medium increased by 50% compared with potato pomace medium (data not shown), which may be one of the reasons accounts for the lower pigment production capacity of hydrolysate, since it was reported that the ethanol produced when glucose used as carbon source could lead to the repression on pigment formation (Chen & Johns, 1994; Tan et al., 2014). Unlike the hydrolysate, when the potato pomace was directly used as substrate, *Monascus purpureus* should first use amylolytic enzyme to hydrolyze starch to produce reducing sugar, so the concentrations of glucose and other monosaccharides had little change during fermentation, which could be in favor of improving the production of pigments and decreasing ethanol production. In addition, the biomass of hydrolysate (9.4 g/L) presented a decrease by 27.1%. The results indicated that the microbial inhibitory compounds derived during hydrolysis process, such as furfural, restrained the key enzyme activities of mycelia growth and metabolism, which would be another reason (Liu et al., 2020).

**TABLE 1:** Comparison of the production of pigments between potato pomace and its hydrolysate

Substrate	Amount	Consumption	Time	Pigments (OD units/ml)				Yield of total pigments	Ratio of red to yellow	Biomass (g/L)	Citrinin (mg/L)
	(g/L)	(g/L)	(day)	Red	Yellow		Total	(OD units/g)			
Potato pomace	40 ± 1.0 (Pomace)	ND	7	27.8 ± 0.8		19.7 ± 1.1	47.5 ± 2.0	1,187.5 ± 13.2^a^	1.41 ± 0.01	12.9 ± 1.2	1.27 ± 0.06
Hydrolysate	40 ± 1.3 (Sugar)	35.4 ± 1.1	7	12.2 ± 1.9^*^		16.5 ± 0.9^*^	28.7 ± 1.9^*^	810.7 ± 11.0^*^	0.72 ± 0.02^*^	9.4 ± 0.8^*^	1.64 ± 0.09^*^

Note

Lower case * indicates the differences are significant or not at the 0.05 level compared with the data obtained under “Potato pomace substrate.” ND indicates that the consumption of pomace was not determined for it was difficult to determine the contents of pomace in the residual. The lower case letters a indicates the yield was calculated on the basis of that 40 g/L of pomace was consumed, the yield should be higher, actually.

Meanwhile, the ratio of red to yellow pigments in the broth of potato pomace was 1.41, and the value of hydrolysate was 0.72 (Table 1), which indicated that the hydrolysate preferred producing yellow pigments and potato pomace preferred producing red pigments. The reason was that variety of nutrients contained in pomace, such as protein and mineral, improved the red pigment production. It supposed that *Monascus* pigments, particularly red pigments, were not formed by biosynthetic pathways only, but by a chemical reaction with compounds containing primary amino groups, such as amino acids and nucleotides (Patrovsky et al., 2019). 100 g of potato pomace contained about 5.2 g of protein (data not shown). Even though the peptone added in medium was the same, the total compounds containing primary amino groups in hydrolysate medium were less than potato pomace medium, because there were few nitrogen sources contained in hydrolysate. Therefore, the pigment production was higher in potato pomace medium and led in particular to the production of red pigments. Citrinin is a mycotoxin produced as secondary metabolite during the *Monascus* pigment production process (Blanc et al., 1995). In view of the harmful of citrinin, the production of citrinin was an important factor to evaluate *Monascus* pigment production process. The results shown in Table 1 indicated that the citrinin produced in potato pomace medium had a decrease of 22.6%, which indicated that potato pomace was a better carbon source to reduce the synthesis of citrinin than its hydrolysate.

It can be seen from above that potato pomace was superior to its hydrolysate when utilized for the production of pigments. Moreover, the hydrolysis process of potato pomace could increase the cost and lower the efficiency. Therefore, only potato pomace, without hydrolysis, was used as substrate in following experiments.

### 3.2 Effect of nitrogen source on pigment production

**FIGURE 2:**
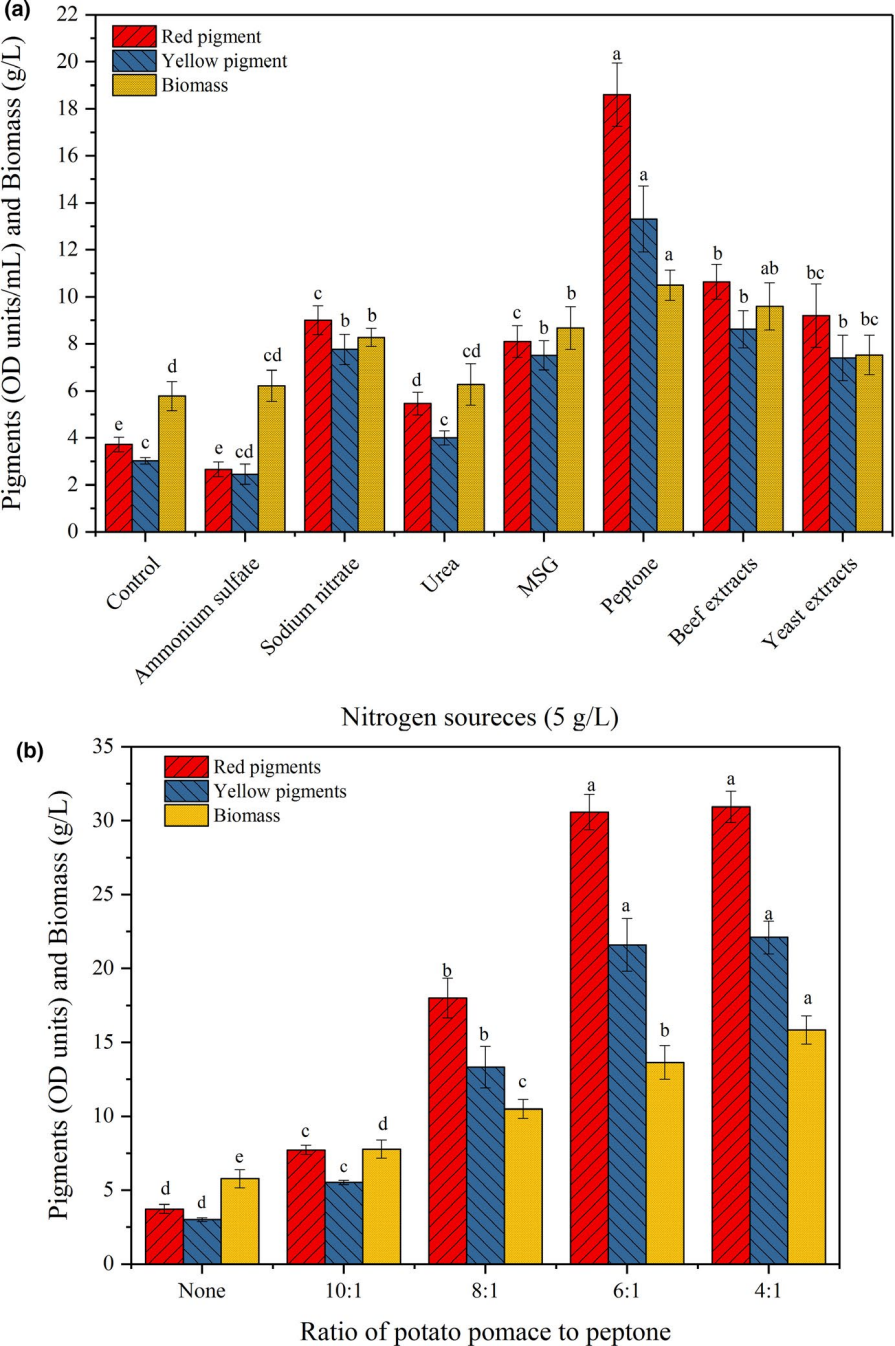
Effect of nitrogen sources on pigment production

It was known that several environmental factors could affect the microbial growth and metabolite production, such as the medium compositions and culture conditions (Babitha et al., 2008; Chen & Johns, 1993). Of which, nitrogen source was an important factor that affecting the growth and pigment production of *Monascus spp*. significantly (Patrovsky et al., 2019; Shi et al., 2015). The results shown in Figure 2a indicated that all nitrogen sources (5 g/L) could promote the growth of mycelia when using potato pomace as carbon source, while the enhancing effects of ammonium sulfate and urea were not distinct (*p* > .05), and others were significant (*p* < .05). By comparison, peptone was more conducive to the growth of mycelia, and the biomass (10.5 g/L) obtained from peptone experiments was nearly 1‐fold higher than the result of the group without any nitrogen sources. Except for ammonium sulfate, other nitrogen sources significantly enhanced the production of pigments (*p* < .05). The results indicated that peptone was a better nitrogen source for pigment production, followed by beef extract. The production of red and yellow pigments in peptone group was 18.6 and 13.3 OD units/ml, which was much higher than other groups. For other microorganisms and carbon sources, monosodium glutamate (MSG) was considered to be a more outstanding nitrogen source for pigment production than peptone (Lin et al., 1992; Wang et al., 2016). Although the pigment production in present study was much higher than the control group, the improving effects on pigment production of MSG were far behind peptone, and even beef extract. Compared with MSG, the total pigments produced from peptone and beef extract increased by 105.5% and 23.1%, respectively. Meanwhile, addition of peptone contributed to red pigment production, and the ratio of red to yellow pigments achieved to 1.4, which was the highest. Consistent with the results in this study, Shi et al. (Shi et al., 2015) also found that peptone could promote the production of extracellular pigments, especially for red pigments, because the presented peptone could enhance the conversion of orange to red pigments, which was due to the increase in available of amino acids. It was believed that peptone could enhance the protein‐bound dissolution of red pigments (Broder & Koehler, 1980). Chen and Johns (Chen & Johns, 1993) found that ammonium salt had similar influences on cell growth and pigment production as peptone and was superior to MSG. The phenomenon was not observed in this study. It was obtained in Figure 2a that the addition of ammonium sulfate generated the increase in biomass, and strangely there were no positive effects on pigment production. It was analyzed that NH_4_^+^ could inhibit some key enzyme activities for pigment synthesis, such as valine dehydrogenase and threonine dehydratase (Zhou et al., 2014).

The effects of different ratios of potato pomace to peptone (C/N mass ratio) on pigment production and mycelia growth were also investigated. The data shown in Figure 2b indicated that the biomass obtained under different C/N ratios presented significant differences (*p* < .05). The maximal biomass (15.8 g/L) was observed at the C/N ratio of 4:1, which had an increase of 16.2% compared with the C/N ratio of 6:1. Surprisingly, the red (30.9 OD units/ml) and yellow pigments (22.1 OD units/ml) produced under the C/N ratio of 6:1 only had an increase of 1.0% and 2.3% compared with the data obtained from the C/N ratio of 6:1, respectively, and the increase was not significant (*p* > .05). However, the addition of peptone at the C/N ratio of 4:1 increased by 50% compared with the C/N ratio of 6:1. Considering the cost of pigment production, the proper C/N ratio for pigment production in this study was determined to be 6:1, which was lower than previous studies (Cho et al., 2002, 2002; Yang et al., 2015), which demonstrated that the process for producing pigments using potato pomace as carbon source was an economic process because of the variety of nutrients contained in pomace.

### 3.3 Effect of initial pH on pigment production

**FIGURE 3:**
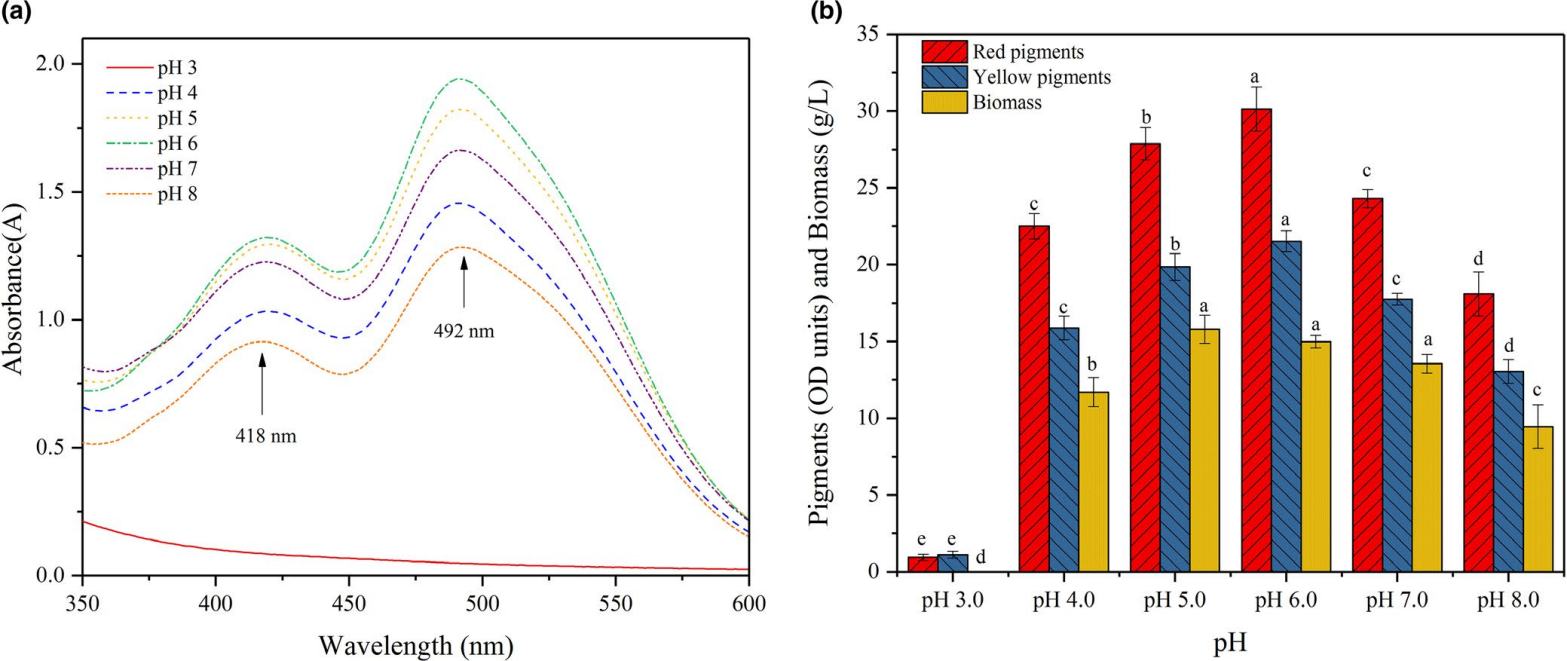
Effect of initial pH on pigment production

Previous studies have revealed that initial pH effectively affected the production and composition of *Monascus* pigments (Chen & Johns, 1993; Patrovsky et al., 2019; Shi et al., 2015). After confirmation of nitrogen source, the effects of initial pH were investigated. In this study, with the exception of pH 3.0, two absorption peaks, representing yellow and red pigments, were observed at 418 and 492 nm for all experimental pH values (pH 4.0–8.0) (Figure 3a). In the experiments of pH 3.0, almost no color was observed. Moreover, all the peaks at 492 nm were higher than the peaks at 418 nm, which demonstrated that the production of red pigments was superior to yellow pigments at pH 4.0–8.0.

The maximum biomass (15.8 g/L) was obtained at pH 5.0. However, the maximal levels of red and yellow pigments were obtained at pH 6.0, which reached 30.1 and 21.5 OD units/ml, respectively (Figure 3b). The red and yellow pigments produced at other pH values were lower than pH 6.0 (*p* < .05). The biomass and pigments at pH 3.0 were difficult to detect, indicating the growth of mycelia was restrained, which was consistent with the scanning spectrum results. However, the results were not agreed with other studies. Patrovsky et al. found that *Monascus purpureus* DBM 4,360 could grow and yield high level of yellow pigments at pH 2.5 (Patrovsky et al., 2019). Another report demonstrated that *Monascus purpureus* KACC 42,430 could tolerate pH 3.0 (Velmurugan et al., 2011). Moreover, previous studies found lower pH was benefit for enhancing yellow pigment production and neutral pH led to red pigment production (Kang et al., 2013; Lee et al., 2007), but the phenomena were not appeared in this study. By calculations, all the ratios of red to yellow pigments at pH 4.0–8.0 were nearly 1.4 (*p* > .05) in this study. The residual solid pomace of pH 6.0 was less than other groups (results not shown). The phenomenon demonstrated that pH 6.0 could only result in a faster fermentation process rather than a redistribution of the intracellular metabolic flux, which may be the reason account for the highest pigment production at pH 6.0, but no obviously changed ratio of red to yellow pigments. The similar results were obtained by Lee et al., and it was demonstrated that *Monascus purpureus* exhibited higher specific substrate consumption rates at pH 5.5 (Lee et al., 2001).

### 3.4 Effect of inoculum size on pigment production

**FIGURE 4:**
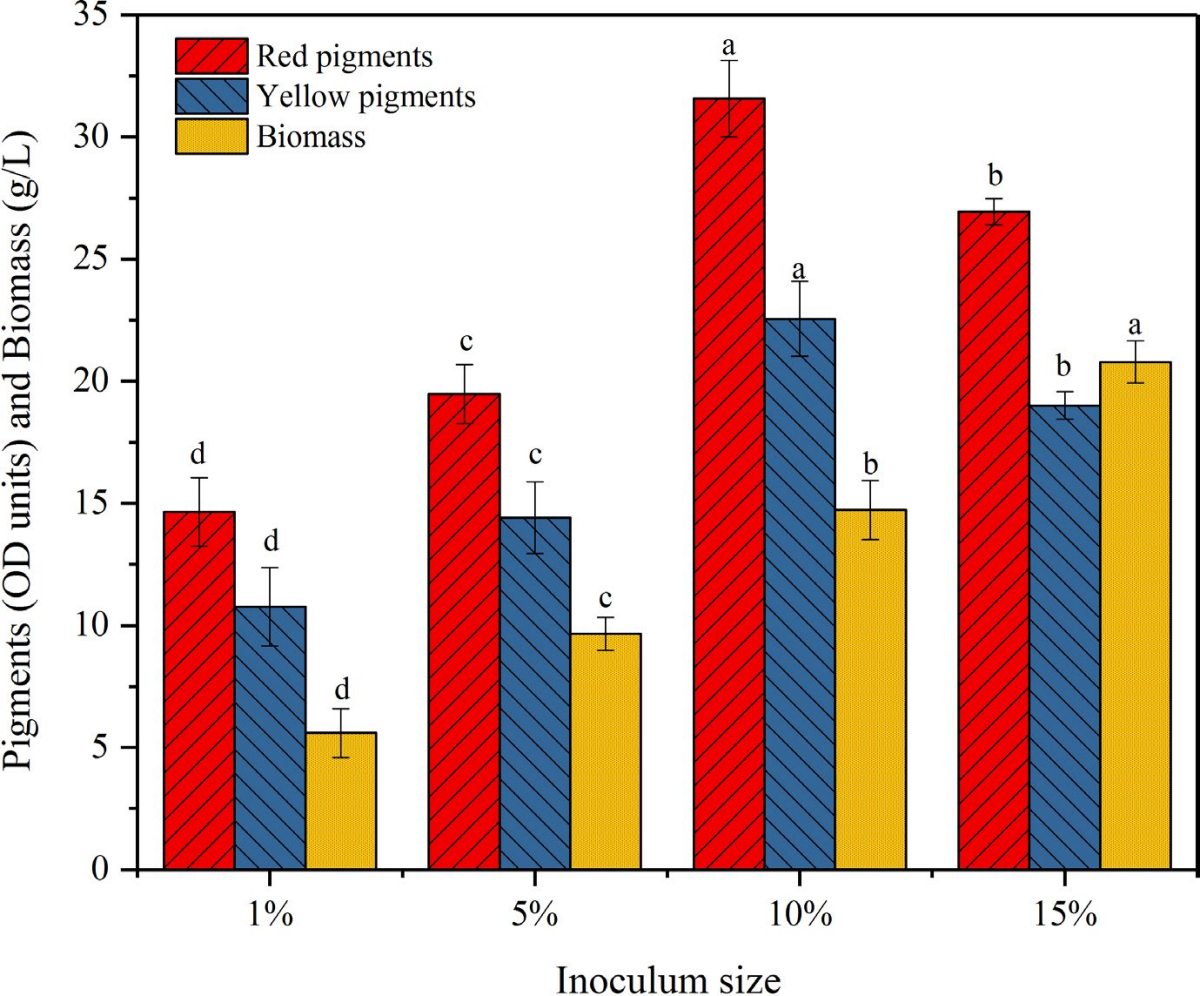
Effect of inoculum size on pigment production

It was reported that different inoculum sizes resulted in different biomass and influenced the fermentation time and efficiency (Velmurugan et al., 2011). The results shown in Figure 4 indicated that biomass was directly proportional to inoculum size, and inoculum size had significant effects on biomass (*p* < .05). The maximal level of biomass (20.9 g/L) was obtained at the size of 15%. Though the biomass obtained from the size of 10% presented a decrease of 28.7% compared with the size of 15%, there was a significant increase in production of red and yellow pigments (*p* < .05), respectively, reached 31.6 and 22.6 OD units/ml. Generally, lower inoculum size led to insufficient mycelia and longer fermentation time, corresponding to a decrease in substrate consumption rate and pigment production efficiency. However, higher inoculation size could cause massive amount of biomass, which resulted in an increase in the viscosity and a decrease in dissolved oxygen of fermentation broth. As a result, the production of pigments was decreased because of the deficient oxygen for metabolism. Besides, more nutrients were consumed for mycelia growth. Only the proper inoculation size could enhance pigment yield and improve the productivity.

### 3.5 Effect of incubation temperature on pigment production

**FIGURE 5:**
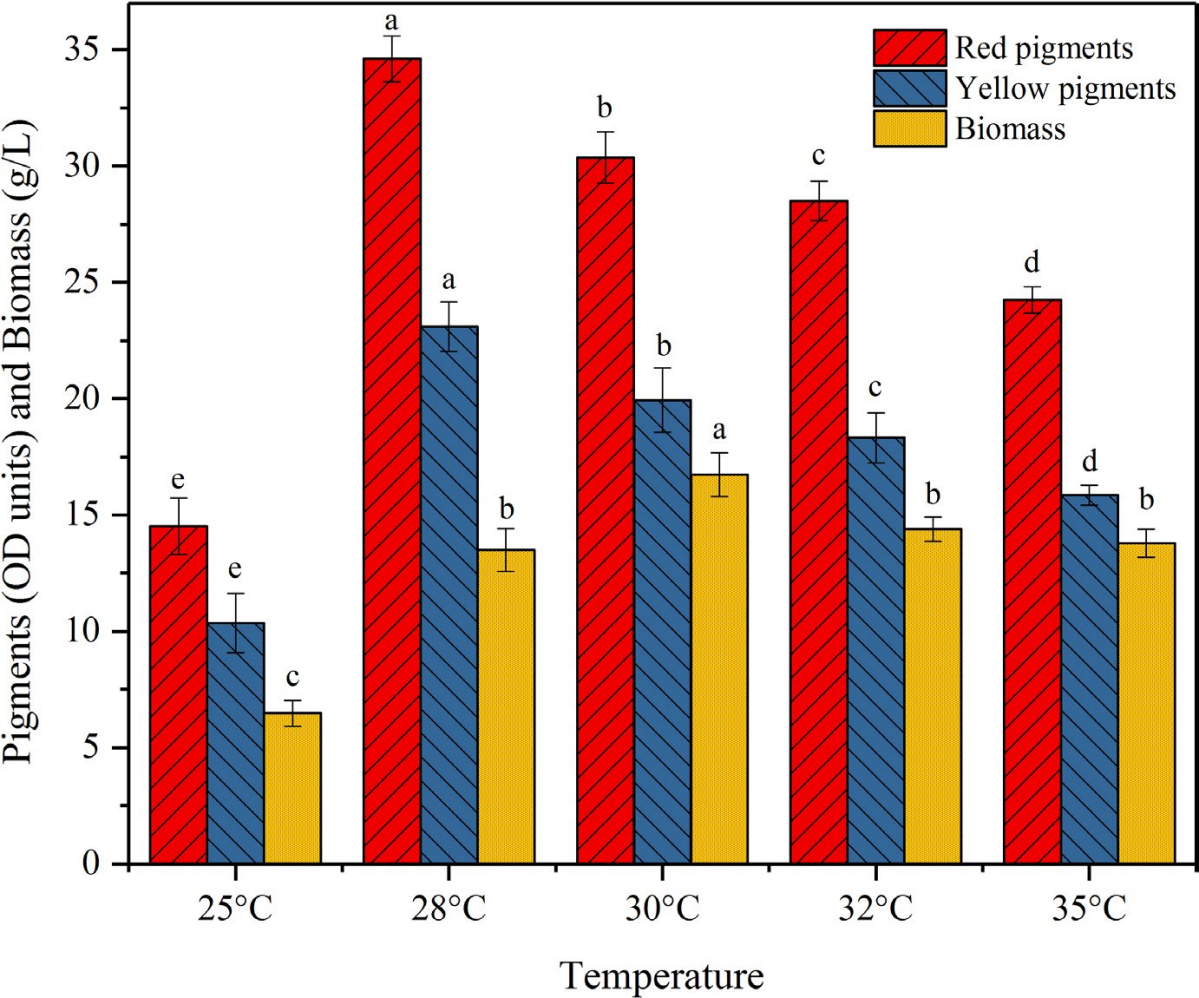
Effect of incubation temperature on pigment production

Because of the mesophilic characteristic of *Monascus spp*., different temperatures could influence the key enzyme activities in mycelia growth and pigment biosynthesis (Chen et al., 2021). It is necessary to search the optimal temperature by experiments for improving pigment production capacity. The maximal level of biomass (16.7 g/L) was obtained at 30℃, indicating that 30℃ was the optimal temperature for mycelia growth when using potato pomace as substrate (Figure 5). Different from biomass, the production of red and yellow pigments reached 34.6 and 23.1 OD units/ml at 28℃, which were the highest and represented an obvious increase than the data of 30℃ (*p* < .05). Moreover, the higher the temperature, the lower the production of pigments (except for 25℃). The results were not agreed with the report of Neera et al. (Dhananjay et al., 2017), who demonstrated that 25℃ was the optimal condition for mycelia growth and pigment production for *Monascus purpureus*. Previous study had reported that the maximum absorption wavelengths of pigments would change when *Monascus purpureus* was incubated at different temperatures (Carvalho et al., 2005), but the phenomena were not observed in this study. Like the results illustrated above, the scanning spectrum under different temperatures presented two absorption peaks at 418 and 492 nm and the ratio of red to yellow pigments had almost no change in this study (data not shown). The above results indicated the types and ratio of pigments produced from potato pomace were relatively stable, which were affected less by pH and temperature.

### 3.6 Batch and fed‐batch fermentations in the 5‐L fermentor

**FIGURE 6:**
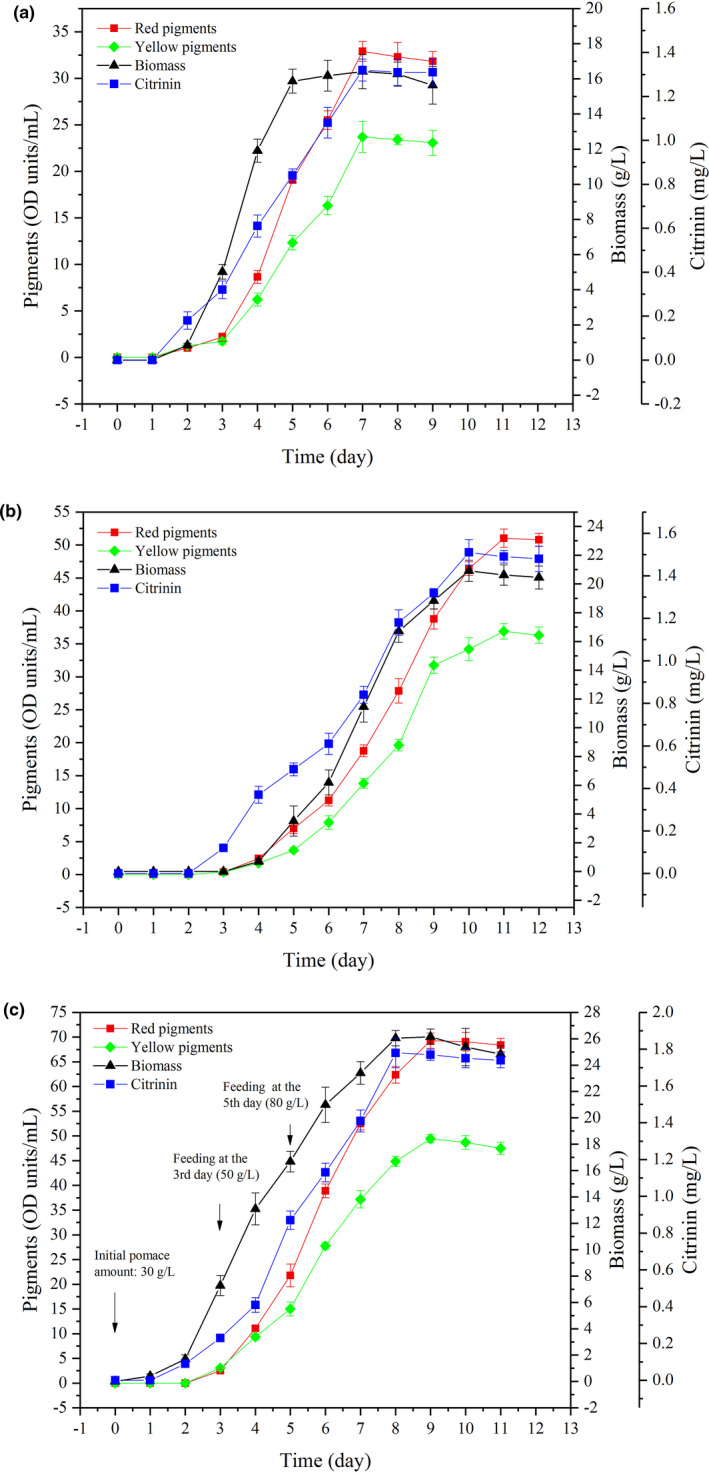
Comparison of the production of pigments in batch and fed‐batch process in a 5‐L fermentor: batch fermentation of 40 g/L potato pomace (a), batch fermentation of 80 g/L potato pomace (b) and fed‐batch fermentation of 80 g/L potato pomace (c)

In this part, the potential ability of pigment production using potato pomace as carbon source was evaluated using a 5‐L tank. Effects of initial amounts of pomace and fed‐batch on pigment production were investigated. The batch fermentation results indicated that mycelia growth presented the model S‐Curve when 40 g/L of pomace was used (Figure 6a). In period of 0‐2nd day, the mycelia started to grow and reached 0.8 g/L, and the mycelia increased quickly in period of 2nd‐5th day. The biomass at the 5th day was 15.9 g/L, only 0.6 g/L less than the maximum obtained at the 7th day (16.4 g/L). The performances of pigment production in 5‐L fermentor were better than the results obtained from Erlenmeyer flask experiments. The maximal levels of red and yellow pigments were observed at the 7th day, which reached 32.9 and 23.7 OD units/ml, with the yield and productivity of total pigments at 1,415.5 OD units/g and 8.2 OD units/(ml·day), respectively (Table 2). After 7 days, the production of pigments began to decline; therefore, the fermentation could be terminated at the 7th day. Different from mycelia growth, the production of pigments increased quickly since the 3rd day. In general, the production process could be divided into three stages: 0‐3rd, 3rd‐7th, and 7th‐9th day. At initial stage (0‐3rd day), there were few pigments produced due to few mycelia biomass. The period of 3rd‐7th day was the main synthesis process: More than 90% of pigments were produced in this period with high production efficiency. In the stage of 7th‐9th day: The production of pigments started dropping slightly. Similarly, other reports found that the pigment production and mycelia growth increased slightly during the initial stage (about 2–3 days ago) and then increased obviously from the 2nd to 4th day (Hu et al., 2012; Yang, Chen, et al., 2015).

**TABLE 2:** Comparison of the batch and fed‐batch fermentations in the 5‐L fermentor

Amount of substrate (g/L)	Time (day)	Total Pigments (OD units/ml)	Biomass (g/L)	Yield of total pigments* (OD units/g)	Productivity of total pigments (OD units/(ml·day))	Citrinin (mg/L)
40	7	56.6 ± 1.5^c^	16.4 ± 1.0^c^	1,415.5 ± 17.2^b^	8.2 ± 0.3^b^	1.32 ± 0.05^c^
80	11	87.9 ± 2.1^b^	20.8 ± 0.6^b^	1,098.3 ± 18.0^c^	7.9 ± 0.1^c^	1.51 ± 0.06^b^
80 (fed‐batch)	9	118.8 ± 2.4^a^	26.2 ± 0.9^a^	1,485.3 ± 16.2^a^	13.2 ± 0.8^a^	1.77 ± 0.03^a^

Note

Single factor analysis of variance was made to find out whether the differences of the three sets of data obtained under different fermentation scales are significant, and lower case b‐c indicates the differences are significantly at the 0.05 level. Mean value with standard deviation shown in the same assay with different lower case letters shows significant differences in each column (*p* < .05). Lower case * indicates the yield was calculated on the hypothesis that all pomace was consumed, the yield should be higher, actually.

When the initial potato pomace was increased to 80 g/L, the mycelia growth also exhibited a model S‐Curve (Figure 6b), but the lag phase was almost 4 days, two days more than the data of 40 g/L. The log phase was also longer, which had 5 days. The phenomena indicated that increasing the amount of pomace could restricted the germination of spore and mycelia growth rate. However, the maximal biomass (20.8 g/L) obtained at the 10th day was higher than the biomass of 40 g/L. Meanwhile, the pigment production had a longer period (4th–11th day), which was almost in synchronization with the mycelia growth. The highest levels of red and yellow pigments were observed at the 11th day, which reached 51.0 and 36.9 OD units/ml, respectively. Although the production of pigments was higher, the yield was at a distinct disadvantage, which had a decrease of 22.4% compared with the data of 40 g/L (Table 2). Moreover, the productivity decreased to 7.9 OD units/(ml·day), which was obviously lower than 40 g/L (*p* < .05). Because the potato pomace was insoluble, it was observed that the viscosity of broth became stickier due to the increased addition of potato pomace, which would result in a decrease of the dissolved oxygen. As a result, the production efficiency was affected because it had been demonstrated that the high dissolved oxygen could improve the pigment production (Pereira et al., 2008). Meanwhile, the mass and heat transfer would be limited by the sticky fluid.

As stated above, increasing the amount of pomace decreased the mycelia growth rate, yield, and productivity of pigments. In order to reduce the disadvantages, fed‐batch experiments were carried out. For fed‐batch experiments, the initial amount of pomace was 30 g/L and increased to 50 and 80 g/L at the 3rd and 5th day by feeding with 300 g/L of potato pomace medium (the C/N was 6:1). It was observed from Figure 6c and Table 2 that fed‐batch process could improve the mycelia growth rate, pigment production yield, and productivities. Although the final amounts of pomace were the same, the production of red and yellow pigments of fed‐batch experiments reached 69.3 and 49.5 OD units/ml, respectively (Figure 6c). The total pigments (118.8 OD units/ml) produced by fed‐batch presented an increase of 35.2% compared with the batch group of 80 g/L; meanwhile, the productivity of total pigments was also improved significantly and had an increase of 67.1% (Table 2). Moreover, the yield in fed‐batch fermentations reached 1,485.3 OD units/g, which was not only much higher than the batch group of 80 g/L, but also significantly increased compared with the data of 40 g/L. The results indicated that fed‐batch process could reduce the inhibition on mycelia growth and pigment production caused by high amount of pomace.

Except for potato pomace, several agroindustrial wasters have been used as substrate to produce pigments by *Monascus purpureus* through submerged process, the production of pigments reported as follows: 0.586 OD units/ml of orange peels in 16 days (Kantifedaki et al., 2018), 18.71 OD units/ml of sugarcane bagasse hydrolysate in 12 days (Hilares et al., 2018), 3.38 OD units/ml of sugarcane bagasse in 14 days (Silveira et al., 2013), 25.8 OD units/ml of corncob hydrolysate in more than 5 days (Zhou, Yin, et al., 2014), 9.06 OD units/ml of grape waste in 9 days (Silveira et al., 2008), and 21.2 OD units/ml of rice straw hydrolysate (Liu et al., 2020). Except for pigments, citrinin was produced as the harmful by‐products. Because of the same precursor (butanone), the production kinetics of citrinin was similar to that of the pigments (Figure 6), and the production of citrinin was proportional to the production of pigments and the amounts of potato pomace (Table 2). The maximal concentration of citrinin was observed in fed‐batch fermentations of 80 g/L, which reached 1.77 mg/L. Although the citrinin produced in fed‐batch fermentations had a distinct increase compared with the results obtained in batch fermentations, it was lower than most of published reports, such as 6.05 mg/L of (Yang, Chen, et al., 2015), 3.0 mg/L of (Min‐Jun, 2009), and 5.5 mg/L of (Orozco & Kilikian, 2008).

By comparison with the results of above studies, it was indicated that potato pomace was the more effective substrate for producing pigments in terms of high pigment production capacity and low citrinin production when the fed‐batch submerged process was used. However, further studies will be designed to improve the production of pigments and reduce the production of citrinin.

## 4 CONCLUSIONS

In order to estimate the practicality of using agroindustrial waste as economical carbon source for producing *Monascus* pigments, the potato pomace and its hydrolysate were first used to compare the pigment production capacity by submerged fermentation, respectively. The results indicated that potato pomace was superior to its hydrolysate for the production of pigments in terms of higher yield, efficiency, and lower cost. Afterward, only potato pomace, without hydrolysis, was used as carbon source to research the optimal pigments producing process conditions. Peptone, pH 6.0, incubation temperature of 28℃, and inoculum size of 10% were in favor of pigment production. High amount of pomace could result in inhibitions on the mycelia growth rate and productivity of total pigments, and fed‐batch process could reduce the inhibitions. With the same final amount of 80 g/L of pomace, the maximal levels of total pigments and productivity produced by fed‐batch process could reach 118.8 OD units/ml and 13.2 OD units/(ml·day), presenting an increase of 35.2% and 67.1% compared with the not fed‐batch group, respectively. In addition, the production of citrinin was at a low level. The results indicated that potato pomace would be a potentially cost‐effective resource for producing *Monascus* pigments through submerged fermentation. Further studies are needed to carry out to investigate the influences of aeration and dissolved oxygen on pigment production efficiency in detail.

## CONFLICT OF INTEREST

The authors declare that they have no competing interests.

## AUTHOR CONTRIBUTION

**Xiaoju Chen:** Conceptualization (lead); Funding acquisition (lead); Investigation (equal); Resources (equal); Supervision (equal); Writing‐original draft (lead); Writing‐review & editing (lead). **Minmin Chen:** Data curation (equal); Formal analysis (equal); Investigation (equal); Methodology (equal); Writing‐original draft (equal). **Xuefeng Wu:** Conceptualization (equal); Funding acquisition (equal); Investigation (equal); Project administration (lead); Resources (lead); Supervision (lead); Writing‐original draft (equal); Writing‐review & editing (lead). **Xingjiang Li:** Data curation (equal); Investigation (equal); Methodology (equal); Resources (equal); Writing‐original draft (equal); Writing‐review & editing (equal).

## ETHICAL STATEMENTS

This study does not involve any human or animal testing.

## References

[fsn32496-bib-0001] Alipour, S. , Habibi, A. , Taavoni, S. , & Varmira, K. (2017). β‐carotene production from soap stock by loofa‐immobilized *Rhodotorula rubra* in an airlift photobioreactor. Process Biochemistry, 54, 9–19.

[fsn32496-bib-0002] Aruldass, C. A. , Dufossé, L. , & Ahmad, W. A. (2018). Current perspective of yellowish‐orange pigments from microorganisms‐ a review. Journal of Cleaner Production, 180, 168–182.

[fsn32496-bib-0003] Babitha, S. , Carvahlo, J. C. , Soccol, C. R. , & Pandey, A. (2008). Effect of light on growth, pigment production and culture morphology of *Monascus purpureus* in solid‐state fermentation. World Journal of Microbiology & Biotechnology, 24, 2671–2675.

[fsn32496-bib-0004] Blanc, P. J. , Laussac, J. P. , Bars, J. L. , Bars, P. L. , Loret, M. O. , Pareilleux, A. , Prome, D. , Prome, J. C. , Santerre, A. L. , & Goma, G. (1995). Characterization of monascidin A from *Monascus* as citrinin. International Journal of Food Microbiology, 27, 201–213. 8579990 10.1016/0168-1605(94)00167-5

[fsn32496-bib-0005] Broder, C. U. , & Koehler, P. E. (1980). Pigments produced by *Monascus purpureus* with regard to quality and quantity. Journal of Food Science, 45, 567–569.

[fsn32496-bib-0006] Carvalho, J. C. D. , Oliva, B. O. , Pandey, A. , & Soccol, C. R. (2005). Biopigments from *Monascus*: Strains selection, citrinin production and color stability. Brazilian Archives of Biology and Technology, 48, 885–894.

[fsn32496-bib-0007] Chen, M. H. , & Johns, M. R. (1993). Effect of pH and nitrogen source on pigment production by *Monascus purpureus* . Applied Microbiology & Biotechnology, 40, 132–138.

[fsn32496-bib-0008] Chen, M. H. , & Johns, M. R. (1994). Effect of carbon source on ethanol and pigment production by *Monascus purpureus* . Enzyme & Microbial Technology, 16, 584–590.

[fsn32496-bib-0009] Chen, W. , Chen, R. , Liu, Q. , He, Y. , He, K. , Ding, X. , Kang, L. , Guo, X. , Xie, N. , & Zhou, Y. (2017). Orange, red, yellow: Biosynthesis of azaphilone pigments in *Monascus* fungi. Chemical Science, 8, 4917–4925. 28959415 10.1039/c7sc00475c PMC5603960

[fsn32496-bib-0010] Chen, X. , Wu, X. , Jiang, S. , & Li, X. (2017). Influence of pH and neutralizing agent on anaerobic succinic acid production by a *Corynebacterium crenatum* strain. Journal of Bioscience & Bioengineering, 124, 439–444. 28583808 10.1016/j.jbiosc.2017.04.021

[fsn32496-bib-0011] Chen, X. , Yan, J. , Chen, J. , Gui, R. , Wu, Y. , & Li, N. (2021). Potato pomace: An efficient resource for *Monascus* pigments production through solid‐state fermentation. Journal of Bioscience and Bioengineering, 132, 167–173. 33941465 10.1016/j.jbiosc.2021.03.007

[fsn32496-bib-0012] Cho, Y. J. , Hwang, H. J. , Kim, S. W. , Song, C. H. , & Yun, J. W. (2002). Effect of carbon source and aeration rate on broth rheology and fungal morphology during red pigment production by *Paecilomyces sinclairii* in a batch bioreactor. Journal of Biotechnology, 95, 13–23. 11879708 10.1016/s0168-1656(01)00445-x

[fsn32496-bib-0013] Cho, Y. J. , Park, J. P. , Hwang, H. J. , Kim, S. W. , & Yun, J. W. (2002). Production of red pigment by submerged culture of *Paecilomyces sinclairii* . Letters in Applied Microbiology, 35, 195–202. 12180940 10.1046/j.1472-765x.2002.01168.x

[fsn32496-bib-0014] Costa, J. P. V. D. , & Vendruscolo, F. (2017). Production of red pigments by *Monascus ruber* CCT 3802 using lactose as a substrate. Biocatalysis & Agricultural Biotechnology, 11, 50–55.

[fsn32496-bib-0015] Dhananjay, K. , Karna, V. R. , & Kumar, S. R. (2017). Optimization of *Monascus* pigment production and its antibacterial activity. International Journal of Current Research in Biosciences and Plant Biology, 4, 71–80.

[fsn32496-bib-0016] Hilares, R. T. , de Souza, R. A. , Marcelino, P. F. , da Silva, S. S. , Dragone, G. , Mussatto, S. I. , & Santos, J. C. (2018). Sugarcane bagasse hydrolysate as a potential feedstock for red pigment production by *Monascus ruber* . Food Chemistry, 245, 786–791. 29287441 10.1016/j.foodchem.2017.11.111

[fsn32496-bib-0017] Hu, Z. , Zhang, X. , Wu, Z. , Qi, H. , & Wang, Z. (2012). Export of intracellular *Monascus* pigments by two‐stage microbial fermentation in nonionic surfactant micelle aqueous solution. Journal of Biotechnology, 162, 202–209. 23079078 10.1016/j.jbiotec.2012.10.004

[fsn32496-bib-0018] Kang, B. , Zhang, X. , Wu, Z. , Qi, H. , & Wang, Z. (2013). Effect of pH and nonionic surfactant on profile of intracellular and extracellular *Monascus pigments* . Process Biochemistry, 48, 759–767.

[fsn32496-bib-0019] Kantifedaki, A. , Kachrimanidou, V. , Mallouchos, A. , Papanikolaou, S. , & Koutinas, A. A. (2018). Orange processing waste valorisation for the production of bio‐based pigments using the fungal strains *Monascus purpureus* and *Penicillium purpurogenum* . Journal of Cleaner Production, 185, 882–890.

[fsn32496-bib-0020] Lee, B. K. , Park, N. H. , Hai, Y. P. , & Chung, W. J. (2001). Production of red pigments by *Monascus purpureusin* submerged culture. Biotechnology & Bioprocess Engineering, 6, 341–346.

[fsn32496-bib-0021] Lee, C. L. , Hung, H. K. , Wang, J. J. , & Pan, T. M. (2007). Improving the ratio of monacolin K to citrinin production of *Monascus purpureus* NTU 568 under dioscorea medium through the mediation of pH value and ethanol addition. Journal of Agricultural and Food Chemistry, 55, 6493–6502. 17636932 10.1021/jf0711946

[fsn32496-bib-0022] Li, L. , Chen, S. , Gao, M. , Ding, B. , & Chen, F. (2019). Acidic conditions induce the accumulation of orange *Monascus* pigments during liquid‐state fermentation of *Monascus ruber* M7. Applied Microbiology and Biotechnology, 103, 8393–8402. 31501941 10.1007/s00253-019-10114-8

[fsn32496-bib-0023] Li, X. , De Ng, Y. , Yang, Y. , Wei, Z. , Cheng, J. , Cao, L. , Mu, D. , Luo, S. , Zhi, Z. , & Jiang, S. (2017). Fermentation process and metabolic fux of ethanol production from the detoxified hydrolyzate of cassava residue. Frontiers in Microbiology, 8, 1603. 28878755 10.3389/fmicb.2017.01603 PMC5572243

[fsn32496-bib-0024] Lin, T. F. , Yakushijin, K. , Büchi, G. H. , & Demain, A. L. (1992). Formation of water‐soluble *Monascus* red pigments by biological and semi‐synthetic processes. Journal of Industrial Microbiology & Biotechnology, 9, 173–179.

[fsn32496-bib-0025] Liu, G. , Xu, Z. , & Cen, P. (2000). A morphologically structured model for mycelial growth and secondary metabolite formation. Chinese Journal of Chemical Engineering, 8, 46–51.

[fsn32496-bib-0026] Liu, J. , Guo, T. , Luo, Y. , Chai, X. , Wu, J. , Zhao, W. , Jiao, P. , Luo, F. , & Lin, Q. (2019). Enhancement of *Monascus* pigment productivity via a simultaneous fermentation process and separation system using immobilized‐cell fermentation. Bioresource Technology, 272, 552–560. 30396112 10.1016/j.biortech.2018.10.072

[fsn32496-bib-0027] Liu, J. , Luo, Y. , Guo, T. , Tang, C. , & Lin, Q. (2020). Cost‐effective pigment production by *Monascus purpureus* using rice straw hydrolysate as substrate in submerged fermentation. Journal of Bioscience and Bioengineering, 129, 229–236. 31500988 10.1016/j.jbiosc.2019.08.007

[fsn32496-bib-0028] Liu, S. , Daigger, G. T. , Kang, J. , & Zhang, G. (2019). Effects of light intensity and photoperiod on pigments production and corresponding key gene expression of *Rhodopseudomonas palustris* in a photobioreactor system. Bioresource Technology, 294, 122172. 31606599 10.1016/j.biortech.2019.122172

[fsn32496-bib-0029] Lv, J. , Zhang, B. B. , Liu, X. D. , Zhang, C. , Chen, L. , Xu, G. R. , & Cheung, P. C. K. (2017). Enhanced production of natural yellow pigments from *Monascus purpureus* by liquid culture: The relationship between fermentation conditions and mycelial morphology. Journal of Bioscience & Bioengineering, 124, 452–458. 28625612 10.1016/j.jbiosc.2017.05.010

[fsn32496-bib-0030] Min‐Jun, X. U. (2009). Ze‐Liang, Yang, Zhi‐Zhou, Liang, Shi‐Ning, and Zhou: Construction of a *Monascus purpureus* mutant showing lower citrinin and higher pigment production by replacement of *ctn*A with *pks*1 without using vector and resistance gene. Journal of Agricultural and Food Chemistry, 57, 9764–9768. 20560630 10.1021/jf9023504

[fsn32496-bib-0031] Nawaraj, G. (2015). Food colorants and their toxicology: An overview. Sunsari Technical College Journal, 2, 69–75.

[fsn32496-bib-0032] Orozco, S. F. B. , & Kilikian, B. V. (2008). Effect of pH on citrinin and red pigments production by *Monascus purpureus* CCT3802. World Journal of Microbiology & Biotechnology, 24, 263–268.

[fsn32496-bib-0033] Patrovsky, M. , Sinovska, K. , Branska, B. , & Patakova, P. (2019). Effect of initial pH, different nitrogen sources, and cultivation time on the production of yellow or orange *Monascus purpureus* pigments and the mycotoxin citrinin. Food Science & Nutrition, 7, 3494–3500. 31763000 10.1002/fsn3.1197 PMC6848812

[fsn32496-bib-0034] Pereira, D. G. , Tonso, A. , & Kilikian, B. V. (2008). Effect of dissolved oxygen concentration on red pigment and citrinin production by *Monascus purpureus* ATCC 36928. Brazilian Journal of Chemical Engineering, 25, 247–253.

[fsn32496-bib-0035] Shahid, M. , Salam, S. , & Mohammad, F. (2013). Recent advancements in natural dye applications: A review. Journal of Cleaner Production, 53, 310–331.

[fsn32496-bib-0036] Shi, K. , Song, D. , Chen, G. , Pistolozzi, M. , & Wu, Z. (2015). Controlling composition and color characteristics of *Monascus* pigments by pH and nitrogen sources in submerged fermentation. Journal of Bioscience & Bioengineering, 120, 145–154. 25648278 10.1016/j.jbiosc.2015.01.001

[fsn32496-bib-0037] Silveira, S. T. , Daroit, D. J. , Anna, V. S. , & Brandelli, A. (2013). Stability modeling of red pigments produced by *Monascus purpureus* in submerged cultivations with sugarcane bagasse. Food & Bioprocess Technology, 6, 1007–1014.

[fsn32496-bib-0038] Silveira, S. T. , Daroit, D. J. , & Brandelli, A. (2008). Pigment production by *Monascus purpureus* in grape waste using factorial design. LWT ‐ Food Science and Technology, 41, 170–174.

[fsn32496-bib-0039] Sun, C. , Wu, X. , Chen, X. , Li, X. , Zheng, Z. , & Jiang, S. (2020). Production and characterization of okara dietary fiber produced by fermentation with *Monascus anka* . Food Chemistry, 316, 126243. 32036177 10.1016/j.foodchem.2020.126243

[fsn32496-bib-0040] Tan, Y.‐Y. , Hsu, W.‐H. , Shih, T.‐W. , Lin, C.‐H. , & Pan, T.‐M. (2014). Proteomic insight into the effect of ethanol on citrinin biosynthesis pathway in *Monascus purpureus* NTU 568. Food Research International, 64, 733–742. 30011710 10.1016/j.foodres.2014.08.004

[fsn32496-bib-0041] Tseng, Y. Y. , Chen, M. T. , & Lin, C. F. (2010). Growth, pigment production and protease activity of *Monascus purpureus* as affected by salt, sodium nitrite, polyphosphate and various sugars. Journal of Applied Microbiology, 88, 31–37. 10.1046/j.1365-2672.2000.00821.x 10735240

[fsn32496-bib-0042] Velmurugan, P. , Hur, H. , Balachandar, V. , Kamala‐Kannan, S. , Lee, K. J. , Lee, S. M. , Chae, J. C. , Shea, P. J. , & Oh, B. T. (2011). *Monascus* pigment production by solid‐state fermentation with corn cob substrate. Journal of Bioscience & Bioengineering, 112, 590–594. 21906997 10.1016/j.jbiosc.2011.08.009

[fsn32496-bib-0043] Vendruscolo, F. , Bühler, R. M. M. , Carvalho, J. C. , Oliveira, D. D. , Moritz, D. E. , Schmidell, W. , & Ninow, J. (2016). L.: *Monascus*: A reality on the production and application of microbial pigments. Applied Biochemistry & Biotechnology, 178, 211–223. 26472672 10.1007/s12010-015-1880-z

[fsn32496-bib-0044] Vendruscolo, F. , & Ninow, J. L. (2014). Apple pomace as a substrate for fungal chitosan production in an airlift bioreactor. Biocatalysis & Agricultural Biotechnology, 3, 338–342.

[fsn32496-bib-0045] Wang, B. , Zhang, X. , & Wu, Z. (2016). Biosynthesis of *Monascus* pigments by resting cell submerged culture in nonionic surfactant micelle aqueous solution. Applied Microbiology & Biotechnology, 100, 7083–7089. 26971494 10.1007/s00253-016-7434-7

[fsn32496-bib-0046] Wang, H. , Ren, Z. , Li, P. , Gu, Y. , Liu, G. , & Jianming, Y. (2011). Improvement of the production of a red pigment in *Penicillium sp*. HSD07B synthesized during co‐culture with *Candida tropicalis* . Bioresource Technology, 102, 6082–6087. 21392975 10.1016/j.biortech.2011.01.040

[fsn32496-bib-0047] Yang, J. , Chen, Q. , Wang, W. , Hu, J. , & Hu, C. (2015). Effect of oxygen supply on *Monascus* pigments and citrinin production in submerged fermentation. Journal of Bioscience and Bioengineering, 119, 564–569. 25488498 10.1016/j.jbiosc.2014.10.014

[fsn32496-bib-0048] Yang, Y. , Liu, B. , Du, X. , Li, P. , Liang, B. , Cheng, X. , Du, L. , Huang, D. , Wang, L. , & Wang, S. (2015). Complete genome sequence and transcriptomics analyses reveal pigment biosynthesis and regulatory mechanisms in an industrial strain, *Monascus purpureus* YY‐1. Scientific Reports, 5, 8331. 25660389 10.1038/srep08331 PMC4321180

[fsn32496-bib-0049] Zhou, B. , Wang, Y. , Lu, H. M. , & Zhou, Y. Q. (2014). Effect of ammonium salts on pigments production by *Monascus anka* mutant in 5L bioreactor. Chiang Mai Journal of Science, 41, 1032–1043.

[fsn32496-bib-0050] Zhou, Z. , Yin, Z. , & Hu, X. (2014). Corncob hydrolysate, an efficient substrate for *Monascus* pigment production through submerged fermentation. Biotechnology & Applied Biochemistry, 61, 716–723. 24673365 10.1002/bab.1225

